# Comparison of screening strategies for prevalent vertebral fractures in South Korea: vertebral fracture assessment vs. spine radiography

**DOI:** 10.1186/s12891-018-1958-1

**Published:** 2018-02-12

**Authors:** Sung-Hee Oh, Dam Kim, Young Eun Lee, Deog-Yoon Kim, Yu Kyung Lee, Joo-Hyun Lee, Sang-Cheol Bae, Yun Young Choi, Junhee Pyo, Jeonghoon Ahn, Yoon-Kyoung Sung

**Affiliations:** 1 National Evidence-based Healthcare Collaborating Agency, Seoul, Republic of Korea; 2 Social and Administrative Pharmacy, College of Pharmacy, Yonsei University, Incheon, Republic of Korea; 3 Department of Rheumatology, Hanyang University Hospital for Rheumatic Diseases, 222-1 wangsimni-ro, Seoundong-gu, Seoul, 133-792 Republic of Korea; 4 Department of Biostatistics and Epidemiology, Graduate School of Public Health, Seoul National University, Seoul, Republic of Korea; 5 Department of Nuclear Medicine, Kyung Hee University Hospital, Seoul, Republic of Korea; 6 Department of Rheumatology, Inje University Ilsan Paik Hospital, Goyang, Republic of Korea; 7 Department of Nuclear Medicine, Hanyang University Hospital, Seoul, Republic of Korea; 8 WHO Collaborating Centre for Pharmaceutical Policy and Regulation, Department of Pharmaceutical Science, Utrecht University, Utrecht, Netherlands; 9 Department of Health Convergence, Ewha Womans University, 52 Ewhayeodae-gil, Seodaemun-gu, Seoul, 03760 Republic of Korea

**Keywords:** Spinal fracture, Radiography, Diagnostic imaging, Cost effectiveness, Radiation

## Abstract

**Background:**

Vertebral Fracture Assessment (VFA) is a useful tool to detect the vertebral fracture (VF) with low cost and radiation exposure. We aimed to compare screening strategies including VFA and spine radiography (X-ray) for detecting VF in terms of clinical effectiveness, cost and radiation exposure.

**Methods:**

Three screening strategies: 1) X-ray following VFA, 2) VFA only, and 3) X-ray only were compared using a Markov model based on administrative data from South Korea in a population aged ≥50 years. We compared the incidence of new VFs, cost-effectiveness of reducing new VFs and radiation exposure in each strategy.

**Results:**

The incidence of new VFs was reduced in all screening strategies compared to no screening: 29.4% for women and 12.5% for men in both X-ray following the VFA and VFA only strategies and 35% for women and 17.5% for men in the X-ray only strategy. The X-ray following VFA strategy had the lowest cost, followed by the X-ray only, and VFA only strategies. The radiation doses for X-ray only were 2,647–2,989 μSv and 3,253–3,398 μSv higher than in the X-ray following VFA and VFA only strategies. The new VF prevention effect was greater in women, and more prominent in older people (women ≥ 70, men ≥ 80) than people ≥ 50 years.

**Conclusions:**

The X-ray following VFA strategy is a cost-effective option for screening prevalent VF to prevent new VF in people aged ≥50 years due to its high effectiveness, lowest cost, and least radiation exposure.

## Background

Vertebral fracture (VF) is the most common sign of osteoporosis and indicates a higher risk of subsequent VFs [1, 2]. According to previous literature, only around one-fourth to one-third of prevalent VFs are recognized clinically [3–5]. However, detection of prevalent VFs is important because new VFs can be prevented by starting osteoporosis medication at the time of detection, even though bone mineral density (BMD) is not low [5–8]. In clinical practice, screening of VFs is rarely performed because of its cost, fear about radiation exposure, and inconvenience.

Spine radiography (X-ray) and vertebral fracture assessment (VFA) by dual energy X-ray absorptiometry (DXA) can readily detect prevalent VFs [9]. Until now, lateral thoracic and lumbar X-rays have been used as the gold standard for VF identification [6], because of the potential false negative rate of VF due to the poor image quality of the upper thoracic vertebrae in VFA [10, 11]. However, VFA has recently been considered as a practical and reliable diagnostic tool with a lower radiation dose of 2–50 μSv compared to 600 μSv for X-rays [12] and provides greater convenience in assessing VFs at the same time as measurement of BMD [5].

Increasing evidence indicates that the sensitivity and specificity of VFA are as high as those of X-ray with lower cost and radiation exposure [13]. There has been one study of the cost-effectiveness of VFA versus X-ray in postmenopausal women with osteopenia [14] but none assessing the clinical benefits and disadvantages of X-ray and VFA as a screening tool in the general population. Therefore, in this study, we aimed to compare the clinical effectiveness, cost-effectiveness, and radiation exposures of VF screening strategies in adults aged 50 years and older using X-ray following VFA, VFA only and X-ray only as performed to detect prevalent VFs early and to prevent new VFs.

## Methods

### Model overview

**Fig. 1:**
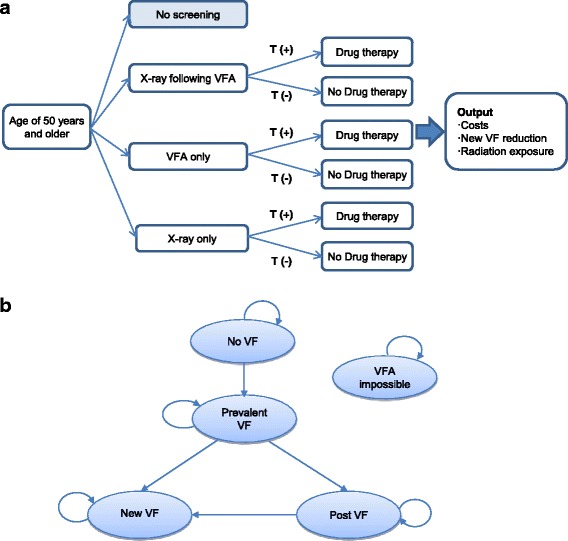
Model structure for screening strategies to identify prevalent VF. T, test; VF, vertebral fracture; VFA, vertebral fracture assessment; (+), positive; (−), negative. **a** Simplified decision tree: Subjects who tested positive for prevalent VF were treated with anti-osteoporotic drugs, and the cycle length of the screening test was two years. b State transition diagram: the Markov model had five health states: No VF, Prevalent VF, Post VF, New VF, and VFA impossible. If the diagnosis of the new VF has been made once, patients with drug therapy are ruled out of the simulation targets

A model structure comparing the screening strategies for prevalent VFs was developed from a review of the literature [15], clinical guidelines [9, 16], and expert opinion. A Markov model using a cohort simulation with each cohort of 1000 samples for women and men aged 50 years and older that compared screening strategies for identifying the presence of both asymptomatic and symptomatic VFs was created. We ran three Markov models simultaneously, one for each of the screening strategies for detecting prevalent VFs: 1) X-ray following VFA, which is screening of VFA followed by X-ray as confirmation test in patients who were suspicious of vertebral fracture, 2) VFA only, and 3) X-ray only. This index strategy of screening was compared to not doing any screening strategy before recognition of a new VF (No screening) (Fig. 1a).

The health states used in the Markov model were *No VF, Prevalent VF, Post VF, New VF*, and *VFA impossible*. *Prevalent VF* included radiographic VFs diagnosed by X-ray as well as symptomatic VFs. The subjects started at an initial state of *No VF* or *Prevalent VF*. We assumed that the patients with a VF could not move back to the *No VF* state. All patientswho tested positive for VF were assumed to receive anti-osteoporotic therapy for one year, and patients who tested negative for VF were assumed not to receive anti-osteoporotic therapy. They could either remain in the present state or transit to *Post VF* or *New VF* in a subsequent cycle depending on the effect of drug therapy. Throughout the 10-year period, each subject was allowed to experience a new VF after the first VF. Once diagnosis of a new VF was made, a patient on drug therapy was excluded from the simulation. The model also considered *VFA impossible* as the absorbing state, which means that the VFA was unreadable as a result of poor image quality (Fig. 1b) [11].

### Probability data and key assumptions

The prevalence of VFs in adults aged 50 years and older was obtained from a large-scale cohort study conducted in South Korea [17, 18]. The incidence of VFs was estimated based on the linked data between a hospital cohort and the National Health Insurance (NHI) claims database of South Korea [19]. Moreover, the adjusted value was multiplied by an asymptomatic to symptomatic VF ratio of 2.8, which was the weighted average calculated by the literature [4, 20, 21]. This was used because the incidence of VFs before adjustment only considers patients who are clinically diagnosed VFs (Table 1).

**Table 1:** Summary of the input parameters in the model for base case analysis and univariate sensitivity analysis

Parameter	Value^a^		Sources	Note
	Women	Men		
Probabilities				
VF prevalence	0.22 [0.08]	0.11 [0.03]	HIRA, [17, 18]	NHI database [Community-based cohort]
two-year VF incidence	0.12 [0.13]	0.05 [0.05]	HIRA, [32]	NHI database [Community-based cohort]
two-year new VF incidence	0.22 [0.23]	0.22 [0.23]	[19, 32]	Hospital cohort- NHI database linked data [Community-based cohort]
RR of new VF on drug therapy	0.58 [0.64]	0.38 [0.41]	[19, 23–26]	Meta-analysis literature review, pooled RR [decrease by 10%]
Diagnostic accuracy				
Sensitivity of X-ray	1.00	1.00		Gold standard
Specificity of X-ray	1.00	1.00		Gold standard
Sensitivity of VFA	0.82 [0.74]	0.82 [0.74]	[22]	SR [95% CI lower limit, conservative]
Specificity of VFA	0.93 [0.89]	0.93 [0.89]	[22]	SR [95% CI lower limit, conservative]
VFA impossible	0.03 [0.05]	0.03 [0.05]	[22]	SR, per patient [per vertebrae]
Costs (€)^b^				
VFA^c^	17 [40]	17 [40]	[27]	50% of X-ray test costs [Increase by 200% of X-ray costs, conservative]
X-ray^c^	24 [17]	24 [17]	HIRA	EDI code, thoracic and lumbar spine in the AP and lateral
VFs treatment	1526 [1249]	1707 [1336]	HIRA	NHI database, per patient [Mild VF relatively]
Physician visits	9	9	HIRA	NHI database, per visit
Drug therapy	180	194	HIRA	Drug weighted average charge, one-year administration
Procedure	809	934	HIRA	NHI database, only in symptomatic VF cases
Radiation doses (μSv)				
X-ray	600	600	[12]	
VFA	25 [2]	25 [2]	[12]	Average level of 2 to 50 μSv [lower limit]

*VF* vertebral fracture, *HIRA* Health Insurance Review and Assessment Service, *NHI* National Health Insurance, *RR* relative risk, *AP* anterior-posterior, *VFA* vertebral fracture assessment, *SR* systematic review

^a^Value in brackets, []: Inputs data for univariate sensitivity analysis

^b^Korean won converted to euros (€) using an exchange rate of 1€ = 1482 KRW (2013)

^c^VFA and X-ray costs included the test and a physician visit

In regard to diagnostic accuracy of VFA, the sensitivity and specificity VFA per-patient were taken from a published systematic review and report [13, 22]. In addition, we calculated the unreadable vertebrae on the VFA as 0.03 (95% CI, 0.04–0.17) per patient and 0.05 (95% CI, 0.04–0.08) per vertebra using random-effect estimates [22]. We assumed that the X-ray has a sensitivity and specificity of 1.00 as the gold standard (Table 1).

The relative risk (RR) for new VFs was derived from five systematic review studies [19, 23–26]. The pooled RR was calculated by averaging the RRs weighted by the market share of the prescribed drug based on the 2012 claims database by health insurance review and assessment service (HIRA) (Table 1). We assumed that patients with diagnosed VFs will receive pharmacotherapy for a one year treatment period according to a previous trial [7] and expert opinions. However, the model did not consider the likelihood of adverse drug reaction from unnecessary treatment with a false-positive VFA image.

### Cost and radiation exposure data

Cost data were collected from the NHI claims data from the HIRA in South Korea [22]. The unit costs for two categories, screening tests and VF treatment, are presented in Table 1. When estimating the costs of the index tests, X-ray was defined as using standard radiographic techniques for VF identification [6]. Since we do not have determined cost of VFA, we calculated that VFA imaging would cost half that of the X-ray, reflecting the U.S. relative cost [27]. The treatment cost of VFs included medical costs such as physician visits, drug therapy, or related procedures. A discount rate of 5% was applied to the future costs with half-cycle corrections according to the South Korean economic evaluation guidelines [28]. All costs were estimated in year 2013 euros using the health care component of the Consumer Price Index [29] and the exchange rate (1€ = 1482 KRW in 2013).

We applied the exposure to radiation at every cycle length of the test at 600 μSv for X-ray; 25 μSv─ middle level of the reported range (2–50 μSv)─for VFA; and 625 μSv for X-ray following VFA, respectively [12]. The strategy of no screening was assumed to have no significant exposure to radiation.

### Statistical analysis

In the base case analysis, we compared the clinical benefits and harm of doing the screening strategies (defined as Do screening, which presents the average expected values of the X-ray following VFA, VFA only, and X-ray only strategies) every two years and No screening. The outcome measures included incidence of new VFs (%), costs of tests and VFs treatment (€), and radiation exposure (μSv) of the three screening strategies compared to the No screening strategy. Consequently, the results were calculated as follows.

$$ \mathrm{Incremental}\ \mathrm{effectiveness}\ \left(\Delta \mathrm{E}\right)={\mathrm{Effect}}_{\mathrm{index}\ \mathrm{test}}\hbox{--} {\mathrm{Effect}}_{\mathrm{no}\ \mathrm{screening}}\ \left(\%\right) $$

$$ \mathrm{Incremental}\ \mathrm{costs}\ \left(\Delta \mathrm{C}\right)={\mathrm{Costs}}_{\mathrm{index}\ \mathrm{test}}\hbox{--} {\mathrm{Costs}}_{\mathrm{no}\ \mathrm{screening}}\ \left(\text{\EUR} \right) $$

$$ \mathrm{Incremental}\ \mathrm{radiation}\ \mathrm{exposure}\ \left(\Delta \mathrm{RE}\right)={\mathrm{Radiation}\ \mathrm{doses}}_{\mathrm{index}\ \mathrm{test}}\hbox{--} {\mathrm{Radiation}\ \mathrm{doses}}_{\mathrm{no}\ \mathrm{screening}}\ \left(\upmu \mathrm{Sv}\right) $$

^*^ Index test: X-ray following VFA, VFA only, and X-ray only as the Do screening.

Next, we performed univariate and multivariate sensitivity analyses to probe the validity of our model. In the univariate sensitivity analyses, the parameters of the prevalence and incidence of VFs, efficacy of drugs, diagnostic accuracy of VFA, costs of test and VFs treatment, and radiation doses of VFA were varied, using the various sources, range, and conservative approach (Table 1). Since there was no recommended test interval available in the guidelines [16], multivariate sensitivity analysis was conducted using a cycle length of one year.

Finally, the subgroup analysis of old age was conducted for women aged 70 years and older and all men aged 80 years and older since these age groups are recommended especially for VFA in the guidelines [16, 30], for which the input parameters are presented in Table 2.

**Table 2:** Summary of the input parameters in the model for multivariate sensitivity analysis and subgroup analysis

Parameters	Value^a^		Sources	Note
	Women	Men		
Cycle length one-year				
one-year VF incidence	0.06	0.02	HIRA	NHI database
one-year new VF incidence	0.11	0.11	[19]	Hospital cohort-NHI database linked data
VFs treatment cost^a^ (€)	1458	1509	HIRA	NHI database
Women aged 70 and older, men aged 80 and older				
VF prevalence	0.43	0.46	[18]	Community-based cohort
two-year VF incidence	0.29	0.17	HIRA	NHI database
two-year new VF incidence	0.32	0.32	[19]	Hospital cohort-NHI database linked data
RR of new VF on drug therapy	0.60	0.60	[33]	Meta-analysis, pooled RR
Sensitivity of VFA	0.88	0.88	[34]	Diagnostic accuracy study for elderly adults
Specificity of VFA	0.99	0.99	[34]	Diagnostic accuracy study for elderly adults
VFs treatment cost^a^ (€)	1513	1675	HIRA	NHI database

*HIRA* Health Insurance Review and Assessment Service, *NHI* National Health Insurance, *VF* vertebral fracture, *VFA* vertebral fracture assessment, *RR* relative risk

^a^Korean won converted to euros (€) using an exchange rate of 1€ = 1482 KRW (2013)

All the statistical analyses were performed by SAS software version 9.2 (SAS Institute Inc., Cary, NC, USA). The model was estimated using TreeAge Pro 2013 (TreeAge Software, Williamstown, MA).

## Results

### Base case analysis

#### No screening versus do screening for prevalent VF

For women aged 50 and over for a 10-year time horizon, the new VF incidence was 54.6% for No screening, and 23.3% for Do screening with a two-year interval (calculated as the average incidence of X-ray following VFA, VFA only, and X-ray only strategies: 25.2%, 25.2%, and 19.6%, respectively). The expected costs were €967 higher for Do screening; and the radiation exposure was 1427 μSv per capita for Do screening. For men aged 50 and over it was also associated with lower new VF incidence (8.4% for Do screening vs. 22.5% for No screening) and higher costs (€658 for Do screening vs. €27 for No screening). Comparing Do screening to identify a prevalent VF to No screening, the new VF incidence decreased by 23.3% with increased costs of €810, and radiation exposure of 1422 μSv for both women and men aged 50 and over as the weighted average by the 2013 registration population by gender based on the South Korean Statistical Information Service statistics (Table 3) [29].

**Table 3:** Results of the base case analysis and multivariate sensitivity analysis for the diagnostic strategies during a 10-year period

Category	Diagnostic strategies	Effectiveness (%)	ΔE^a^ (%)	Costs (€)	ΔC^b^ (€)	ΔRE^c^ (μSv)
Women						
Base case	No screening	54.6		60		
	Do screening^e^	23.3	−31.3	1027	967	1427
	X-ray following VFA	25.2	−29.4	881	821	747
	VFA	25.2	−29.4	1202	1142	141
	X-ray	19.6	−35.0	998	938	3394
Multivariate sensitivity (Cl = 1 year)	No screening	54.6		60		
	Do screening^e^	23.2	−31.4	1102	1042	2455
	X-ray following VFA	24.8	−29.7	862	802	996
	VFA	24.8	−29.7	1418	1358	254
	X-ray	19.9	−34.7	1025	966	6115
Men						
Base case	No screening	22.5		27		
	Do screening^e^	8.4	−14.2	658	630	1416
	X-ray following VFA	10.1	−12.5	504	477	556
	VFA	10.1	−12.5	927	899	147
	X-ray	5.0	−17.5	542	515	3545
Multivariate sensitivity (Cl = 1 year)	No screening	54.6		60		
	Do screening^e^	8.3	−14.2	734	707	2526
	X-ray following VFA	10.1	−12.4	498	471	828
	VFA	10.1	−12.4	1143	1116	267
	X-ray	4.8	−17.7	560	533	6483
Total^d^	Do screening^e^	16.4	−23.3	854	810	1422

*VFA* vertebral fracture assessment, *Cl* Cycle length

^a^ΔE (Incremental effectiveness, %) = Effect_index test_–Effect_no screening_ in new VFs incident

^b^ΔC (Incremental Costs, €) = Costs_index test_–Costs_no screening_ in the costs of test and VFs treatment

^c^ΔRE (Incremental radiation exposure, μSv) = Radiation doses_index test_–Radiation doses_no screening_; Radiation doses_no screening_ was assumed to be ‘0 μSv’

^d^In the base case, the weighted average by the registration population by gender (female = 8,649,974 people; male = 7,590,057 people) based on Statistics

^e^Do screening presents the average expected values of X-ray following VFA, VFA only, and X-ray only

#### Three screening strategies for prevalent VF

Among the three screening strategies performed every two years (Table 3), the X-ray following VFA and VFA only strategies showed the same clinical effect of reducing the new VF incidence by as much as 29.4% for women, and 12.5% for men. The most effective strategy was the X-ray only strategy with a new VF incidence reduction of 35% for women and 17.5% for men over a 10-year period. With regard to the costs, X-ray following VFA strategy was less expensive than the others for both women and men. The second economic option was X-ray only strategy, then VFA only strategy (ΔC of €821, €938, and €1142 for women; €477, €515, and €899 for men per capita, respectively). Also, the 10-year radiation doses in the X-ray only strategy was from 2647 to 2989 μSv and 3253 to 3398 μSv higher than in the X-ray following VFA or VFA only strategies for both women and men.

### Sensitivity analysis

**Fig. 2:**
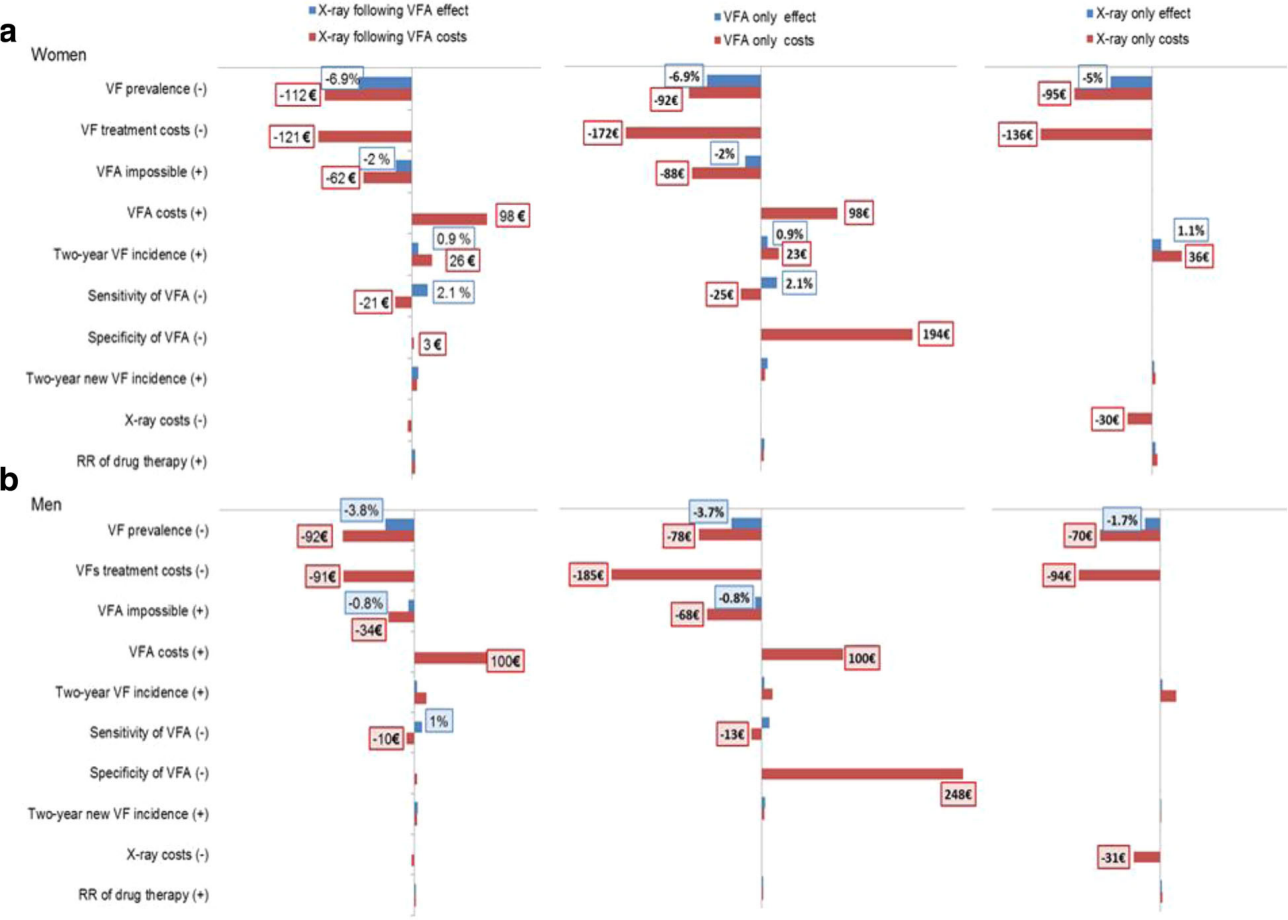
VF, vertebral fracture; VFA, vertebral fracture assessment; RR, relative risk; (+), increase; (-), decrease (refer to Table 1). Effect and impact of parameter variation on new VF incidence compared with the base case analysis; Costs and impact of parameter variation on costs compared with the base case analysis. (**a**) represent for women and (**b**) for men

The results of the univariate sensitivity analysis compared with the base case analysis are shown in Fig. 2. The input parameters of the sensitivity analysis are given in Table 1. The decrease of VF prevalence indicates that not only was the new VF incidence lower, by 1.7% to 6.9%, but the expected cost was reduced by €70 to €112 in all the screening strategies for both women and men. A similar pattern was found for VF incidence, as reduced effectiveness and cost resulted from an increase in VF incidence. Even though these parameters made great changes in effectiveness and cost, the relative rankings among the strategies did not change. In addition, when the sensitivity and specificity of VFA were reduced, the costs of the strategies that included VFA changed. However, the relative rankings of the strategies were maintained.

The multivariate sensitivity analysis examined the influence of a one-year cycle length compared to the base case analysis of a cycle length of two years during a 10-year period (Table 3). In particular, the X-ray following VFA strategy performed every year for women was beneficial as it was associated with an effect increase in terms of new VF incidence reduction, cost savings, and a slight increase in radiation dose, whereas the X-ray only produced the opposite effect of the X-ray following VFA strategy. The results of the X-ray following VFA strategy for men were similar regardless of the cycle length and the X-ray only strategy of every year increased the effectiveness, cost, and radiation dose. On average, the effectiveness of Do screening on the reduction of new VF incidence was almost identical between the one- and two -year screen intervals. However, the expected cost (about €80 in women and men) and the radiation exposure (about 1000–1100 μSv in women and men) were slightly increased in the one-year cycle length.

### Subgroup analysis

**Fig. 3:**
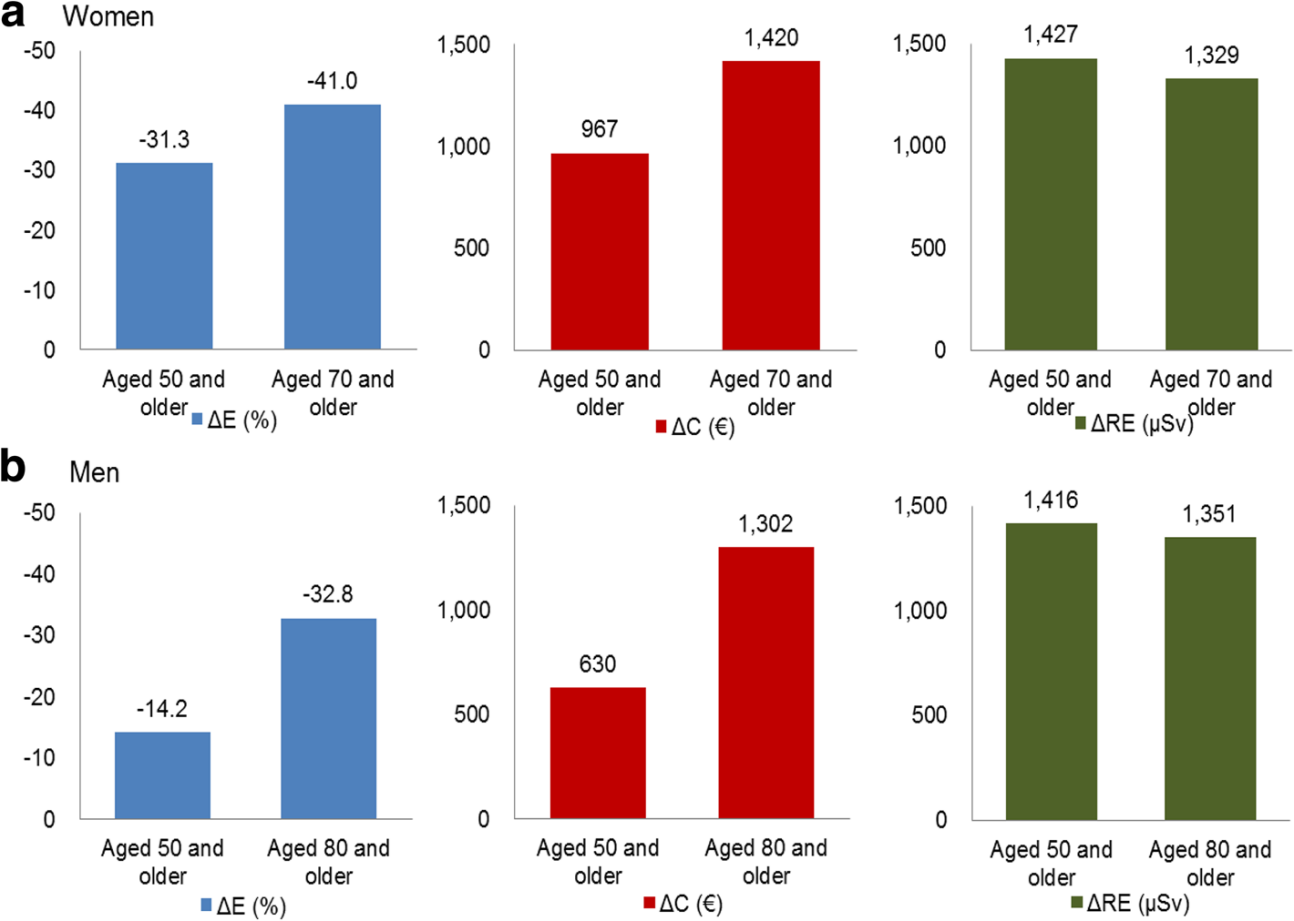
Subgroup analysis by old age people who had received prevalent VF screening strategies compared to No screening in a 10-year time horizon. ΔE, new VFs incident reduction; ΔC, increased costs; **Δ**RE, radiation exposure. VF, vertebral fracture; VFA, vertebral fracture assessment. **a** and **b** represent the average values of the X-ray following VFA, VFA, and X-ray as a Do screening strategy

Figure 3 shows the results from the subgroup analysis for old age people; women aged 70 and older and men aged 80 and older. The preventive effect of Do screening on new VFs was 41.0% for women and 32.8% for men compared with No screening. The expected costs were €1602 per woman aged 70 and over, and €1429 per man aged 80 and over. The radiation exposure for the old age women and men was comparable with the base case results. Among the three strategies, the overall expected cost of X-ray only strategy was the highest in the subgroup for old people whereas the cost of VFA only strategy was the highest in the base case analysis (Data not shown).

## Discussion

This study demonstrated that doing screening strategies for prevalent VFs has a high preventive effect on reducing new VF incidence for women and men aged 50 years and older compared to a No screening strategy. A cost-effective option was X-ray following VFA strategy, considering that the radiation exposure in X-ray only strategy was much higher than the X-ray following VFA or VFA only strategies.

For a change of two years into the one-year screening interval, the average effects of the three screening strategies for prevalent VF on reducing new VF incidence were almost identical, but the X-ray following VFA strategy for women was especially beneficial resulting in a reduction of the incidence of new VFs, cost savings, and relatively low levels of radiation dose. On average, the clinical effect of incident VF reduction was more than two times in women than in men, more in old people aged 70 and over than in people aged 50 and over, and its effect on old people was increased more in men than in women.

In clinical practice, the X-ray, the gold standard tool for detecting VF, is not routinely performed in the clinical evaluation of people at risk of future fractures. In our study, considering the highest radiation exposure and the second highest cost, a routine X-ray screening strategy might be inferior to the X-ray following VFA. Given that the worldwide average effective dose from natural background radiation was reported to be 2.4 mSv per year [12], the potential risk to an individual might be small because the annual radiation doses of the X-ray strategy in our study had a much lower dose at 0.35 mSv. However, cyclical use could have a psychological impact on the compliance of people who are eligible for routine screening for VF.

On the other hand, VFA had the lowest radiation dose and a high preventive effect of new VFs. However, we should carefully consider whether to replace the X-ray with VFA because performing the VFA has a risk of misdiagnosis caused by false negative rates of about 20% [10]; the costs of VFA are increased with the additional costs of patients receiving unnecessary treatment with a false-positive result. Moreover, even if most osteoporotic VFs occur between T7 and L3, the poor image quality of the upper thoracic vertebrae superior to T7 is a major limitation of VFA [11]. Thus our model applied the unreadable probability of VFA, and additional confirmatory imaging (e.g. X-ray) should be considered.

The X-ray following VFA strategy can overcome these problems of VFA with the lowest cost, lower radiation dose than X-ray, and high effect of reducing the new VF. In addition, it can assess VFs at the same time as BMD assessment in contrast to X-ray, which may need referral to another facility [8].

Our study has some limitations. First, since there were no reliable data about adherence to screening and osteoporosis medication, we assumed 100% adherence for screening as well as medication use. Therefore, this assumption probably overstates the clinical effectiveness of this study. In addition, the use of anti-osteoporotic agents based on BMD values was not considered, since it would be equally applicable to all the patients. Modeling that included information on BMD values in the subject population would be very complex, but it would be possible to perform such additional modeling if real world data from observational studies were included. Second, as we assume that the costs of the VFA test would be half of the X-ray as in the U.S., it should be updated for costs after pricing in each countries. Third, our analysis did not consider patient outcome or health-related quality of life (HRQoL). However, this study has great strength that it revealed the benefits and harm of X-ray following VFA, VFA only, and X- ray only strategies, as a population screening tool. Moreover, we used accurate data on prevalence, incidence and cost using real world data from the NHI claims database to obtain a more accurate result. Although the new DXA devices such as iDXA are not widely used in the clinical field, the results of this study would be different if we used such a device because of its high sensitivity and specificity [31].

## Conclusion

We suggest that routine screening for VF every one to two years is justified in all populations aged 50 years and older. In particular, an X-ray following VFA strategy can be a relevant option for prevalent VF detection and new VF prevention. Further studies of both HRQoL and cost-utility analysis are needed to promote informed decision making.

## Abbreviations

BMD: Bone mineral density
DXA: Dual energy X-ray absorptiometry
HIRA: Health insurance review and assessment service
HRQoL: Health-related quality of life
NHI: National Health Insurance
RR: Relative risk
VF: Vertebral fracture
VFA: Vertebral fracture assessment
X-ray: Spine radiography
